# Open distal anastomosis technique in acute type A aortic dissection

**DOI:** 10.1186/1749-8090-8-S1-O15

**Published:** 2013-09-11

**Authors:** D Cvetkovic, M Kocica, Lj Soskic, B Milicic, N Aleksic, M Ristic

**Affiliations:** 1 Clinic for Cardiac Surgery, UC Clinical Centre of Serbia, Belgrade, Serbia; 2 Clinic for Cardiology, UC Clinical Centre of Serbia, Belgrade, Serbia

## Background

Acute ascending aortic dissections is the most common catastrophe of the aorta that requires emergency surgery. There is still a debate regarding the optimal surgical approaches for construction of the distal anastomosis in replacing the ascending aorta. The purpose of this study was to evaluate early clinical outcomes of two different surgical techniques: open distal anastomosis in hypothermic circulatory arrest (OHCA) compared to anastomosis with clamped aorta while continuing on extracorporeal circulation (CECC).

## Methods

A prospective randomized study in 84 consecutive patients who were operated, between September 2008 and September 2010, for acute type A aortic dissection with isolated replacement of the ascending aorta. The influence of two techniques (OHCA n = 35, CECC n = 49) on clinical outcome was investigated.

## Results

No significant difference was found between the two groups in terms of age and sex distribution. The groups were comparable with respect to all preoperative and intraoperative predictive risk factors for early clinical outcome and complications. The mean period of circulatory arrest lasted 30,46 min (15-61 min) at an average nasopharyngeal temperature of 23.57°C (16-28°C). Treatment method (OHCA vs CECC) was not associated with differences in intrahospital mortality (28.6% vs 26.5%, p=0,836) nore in early major postoperative complications: de novo focal neurolocigal deficits (11.4% vs 10.2%, p=0,858), acute renal failure (8.6% vs 10.2, p=0,802), blood loss (1289.71 ml vs 1126.12 ml, p=0,892) or reexploration for hemorrhage (8.6% vs 8.2%, p=0,947).

## Conclusion

While there is no difference in clinical outcome in surgical treatment of acute type A aortic dissection with or without circulatory arrest, there are some practical technical advantages if the distal anastomosis is performed in an open manner. Probably the long-term outcome would also be better with this anastomosis technique.

